# Participants' experiences of mental health during a COVID-19 tailored ACT-based behavioural weight management intervention: a qualitative study

**DOI:** 10.1080/17482631.2022.2123093

**Published:** 2022-09-13

**Authors:** Rebecca A. Jones, Rebecca Richards, Roshni Palat, Carly Hughes, Andrew J Hill, Ann Vincent, Simon J Griffin, Amy L. Ahern, Robbie Duschinsky

**Affiliations:** a MRC Epidemiology Unit, University of Cambridge, Cambridge, UK; b School of Clinical Medicine, University of Cambridge, Cambridge, UK; c Fakenham Medical Practice, Norfolk, UK; d Norwich Medical School, University of East Anglia, Norwich, UK; e Division of Psychological & Social Medicine, School of Medicine, University of Leeds, Leeds, UK; f Department of Medicine, University College London, London, UK; g Primary Care Unit, Department of Public Health and Primary Care, University of Cambridge, Cambridge, UK

**Keywords:** COVID-19, obesity, mental health, interventions, psychological therapies, weight management

## Abstract

**Purpose:**

We aimed to explore participants' experiences of mental health during an acceptance and commitment therapy (ACT)-based guided self-help intervention to support weight management in adults with overweight or obesity during the COVID-19 pandemic (SWiM-C: Supporting Weight Management during COVID-19).

**Methods:**

We conducted semi-structured telephone interviews with twenty participants and used reflexive thematic analysis to identify patterns of meaning across the dataset relevant to mental health.

**Results:**

Four themes were conceptualized: i) Mental health changes associated with SWiM-C, ii) External factors negatively impacted mental health and intervention engagement, iii) Use and impact of coping responses, and iv) Intervention preferences based on psychological needs.

**Conclusions:**

Findings suggest that participants were exposed to multiple factors, both related to and external to the intervention, that negatively impact their mental health, yet ACT-based aspects of the SWiM-C intervention appeared to support participants to adaptively manage the decline in their mental health. The findings can be used to inform the development of future weight management interventions, such as through intervention personalization and the inclusion of more strategies that target emotional regulation.

**Trial registration**: ISRCTN 12107048, https://www.isrctn.com/ISRCTN12107048

## Introduction

Despite the well-evidenced relationship between obesity and mental health (Bak et al., 2014; Fabricatore et al., 2011; Fruh, 2017; Geiker et al., 2018; McKibbin et al., 2014; World Health Organization, 2013), research investigating behavioural treatments for obesity often still lack sufficient focus on the role of mental health. This was highlighted by a recent systematic review that found a scarcity of high-quality research investigating the impact of behavioural weight management interventions on mental health (Jones et al., 2021). Weight management requires a vast amount of mental and emotional investment, however previous research has found little or no mental health support within weight management services (Kirk et al., 2014). Further research is needed to clarify whether these interventions sufficiently support mental health, and how they can be improved.

On 11 March 2020, the World Health Organization (WHO) declared the novel coronavirus outbreak (COVID-19) to be a global pandemic (World Health Organization, 2020). The COVID-19 pandemic represents a unique period of heightened distress, with research showing that those living with obesity experienced a detrimental impact on their mental health at a greater rate than those without pre-existing conditions (such as obesity) (Brooks et al., 2020; Brown et al., 2021; Choi et al., 2020; Rajkumar, 2020; Shah et al., 2020; Shigemura et al., 2020). The increased risk of poor mental health in adults with obesity during the pandemic amplifies the need for weight management services to provide adequate mental health support. However, the introduction of pandemic restrictions, such as social distancing and isolation measures, resulted in the suspension of many face-to-face weight management services (UK Government, 2020). Alternative methods of remotely providing support were required.

Evidence suggests that behavioural weight management interventions based on acceptance and commitment therapy (ACT) may be more supportive of mental health for adults with overweight and obesity than standard behavioural treatment (Lawlor, Islam et al., 2020). ACT is an action-based approach to behavioural therapy, with a core focus on accepting what is outside of personal control, and committing to changing that which is within personal control (Hayes, 2016). ACT-based interventions are increasingly being provided remotely as “guided self-help”—these are interventions that are predominantly self-directed with periodic support from a trained practitioner (Cavanagh et al., 2014; Epping-Jordan et al., 2016; Purgato et al., 2019; World Health Organization, 2015). Research on ACT-based guided self-help interventions in other domains (e.g., stress management interventions) has shown improved access to hard-to-reach and/or isolated populations, whilst requiring fewer resources and producing similar effects to face-to-face treatment for psychological outcomes (Cavanagh et al., 2014; Epping-Jordan et al., 2016; Purgato et al., 2019; World Health Organization, 2015). However, despite growing evidence for ACT-based weight management interventions, there is currently limited evidence assessing how effectively these interventions support mental health, particularly when delivered remotely as “guided self-help” (Cavanagh et al., 2014; Lawlor, Islam et al., 2020). Understanding the impact of these interventions on mental health, particularly during periods of heightened distress and reduced access to standard support, may inform the development of more psychologically supportive interventions for adults with overweight or obesity.

In response to the need for remote methods of providing psychological support for adults with overweight or obesity, and the growing evidence for ACT-based interventions, we developed the SWiM-C (Supporting Weight Management during COVID-19) intervention. SWiM-C was a web-based, guided self-help, ACT-based intervention that aimed to support adults with overweight or obesity to prevent weight gain and manage their mental health during the COVID-19 pandemic. As the SWiM-C intervention was ACT-based, it centred on core concepts such as mindfulness, acceptance, and values clarification that ultimately aim to enhance psychological flexibility (Hayes, 2016). Psychological flexibility encourages self-compassion and tolerance towards unpleasant thoughts, feelings, and bodily sensations (Hayes, 2016). The SWiM-C intervention included strategies such as present-moment awareness, cognitive defusion, and urge-surfing help to achieve this. Thus, SWiM-C may support participants to better manage the psychological demands of weight management efforts, particularly during the context of the COVID-19 pandemic.

The purpose of this qualitative study was to broadly explore intervention participants’ mental health experiences during the SWiM-C intervention, and how weight management interventions could be adapted to better support mental health. In this study, we embraced the “symptom continuum” definition of mental health throughout the research process. This definition appreciates that individuals can experience one or more symptoms of mental ill-health whilst not meeting diagnostic criteria (Angermeyer et al., 2015; Seow et al., 2017). The symptom continuum permits the exploration of a broad array of mental health-related outcomes, such as mood, stress, loneliness, self-esteem, and more broadly, mental wellbeing.

Findings from this study will inform the development of future weight management interventions, as well as informing how SWiM-C could be adapted and optimized for other contexts. While the pandemic represents a unique situation, findings of this study may be generalizable to weight management interventions in other contexts of high stress, reduced access to support and resources, and high levels of social isolation.

## Methods

This study adheres to the guidelines and recommendations provided by Standards for Reporting Qualitative Research (SRQR) and Consolidated Criteria for Reporting Qualitative Studies (COREQ; O’Brien et al., 2014; Tong et al., 2018).

### Design and setting

#### Study design and setting

This qualitative study was embedded in the SWiM-C (Supporting Weight Management during COVID-19) randomized controlled trial (Clinical trial registration: ISRCTN12107048). Ethical approval was obtained from the Cambridge Psychology Research Ethics Committee (Application No: PRE.2020.049) on 24/04/2020. All participants gave written, informed consent. Participants were randomized to either the SWIM-C intervention or to a standard advice wait-list control with 1:1 allocation. Standard advice was provided in the format of a leaflet with information on diet, physical activity, and mood during the pandemic. Outcome assessments were completed online at baseline and 4-months from baseline. Information was collected on height, weight, demographics, eating behaviour, physical activity, quality of life/wellbeing, and intervention engagement.

#### SWiM-C intervention

SWiM-C was a web-based, guided self-help intervention that aimed to support adults with overweight or obesity with weight management, health behaviours, and emotional wellbeing during the COVID-19 pandemic. The intervention was based on ACT and included 12 weekly sessions delivered online. Sessions covered topics such as control and acceptance, willingness, overcoming obstacles, stress management, and urges and cravings; this list is not exhaustive. Each session consisted of psychoeducation, reflective exercises, and behavioural experiments. The “guided” element of the self-help programme was provided by trained non-specialists (“SWiM Coaches”). SWiM coaches had a 20-minute phone-call with each participant following completion of session 4 and sent a tailored email to the same participant following their completion of session 10. Further information is reported elsewhere (Mueller et al., 2022).

### Participant recruitment and sampling

#### Eligibility criteria

Participants were adults with overweight or obesity (≥18 years, ≥25 kg/m^2^) who had a good understanding of written English and owned a set of bodyweight scales. Participants were excluded from taking part if they had undergone bariatric surgery in the last 2 years. Participants were not excluded based on mental health diagnosis. To be eligible for participation in the embedded qualitative study, participants were required to provide informed consent to be contacted and invited to interview.

#### Participants

Twenty SWiM-C intervention participants were purposively sampled for maximum variation in broad demographic data (age, sex, education, ethnicity, occupation) to ensure study findings represented a diverse range of participants. Participant baseline characteristics are described in Table I.

**Table I.** Baseline characteristics of interview study participants (n = 20).

Characteristic		Number and percentage of participants (unless otherwise stated)
Age (mean [min – max])		52.04 (25-84)
Sex	Male	6
	Female	14
Marital status	Single	7
	Married/Civil partnership	9
	Co-habiting	2
	Widowed	1
	Separated/Divorced	1
Ethnicity	White	14
	Mixed	2
	Black	1
	Asian	1
Education status	No formal qualification	1
	GCSE or equivalent	3
	A-Level or equivalent	6
	Post-secondary education	3
	University Degree or equivalent	4
	Higher Degree or equivalent	3
Occupation status	Employee in full-time job	6
	Employee in part-time job	4
	Self-employed full or part-time	3
	Full-time education at school / college	1
	Unemployed and available for work	1
	Wholly retired from work	5
Body mass index (kg/m^2^) (mean [min – max])		33.34 (25.31–52.13)
Self-reported change in weight from baseline to 4 months	Lost weight	7
	Gained weight	10
	Don’t know	3
Symptoms of depression	None to mild (score of ≤9)	12
	Moderate to severe (score of 10-24)	8
Symptoms of anxiety	None to mild (score of ≤9)	14
	Moderate to severe (score of 10-21)	6
Perceived stress (higher scores represent greater stress) (mean [min – max])		6.55 (3-10)
Number of sessions completed (mean [min – max])		9.3 (2.5 – 12)

The sample size was estimated to be sufficient based on previous relevant studies and the pragmatic restraints of the COVID-19 pandemic (Ahern et al., 2013; Ingels & Zizzi, 2019; Johansson et al., 2015; Lawlor, Hughes et al., 2020; Rand et al., 2017). Interview participants were given a minimum of 48 hours to read the participant information sheet and multiple opportunities to ask questions. Written informed consent was obtained prior to any study activities. Participants received a £20 gift voucher as an honorarium after completing the interview.

### Data collection

Interviews were conducted by a female PhD researcher (RAJ) and a female postdoctoral researcher (RR) with experience in qualitative interviewing and backgrounds in public health, weight management and health psychology. Researchers were not known to the participants prior to taking part in the study. There was no one else present during the interviews, besides the interviewer and participant. Twenty individual semi-structured interviews were conducted at the end of the SWiM-C intervention by telephone. The interview schedule (Supplementary material one: Interview schedule for intervention participants) was developed from previous literature, investigator expertise, and with experts from relevant fields of obesity, psychology, and qualitative research. Interview schedules explored participants’ experiences of the intervention, intervention acceptability (including benefits and disadvantages), and experiences of mental health during SWiM-C. The interview schedule did not explicitly ask pandemic-related questions. The schedule was piloted by RAJ with two patient representatives and revised accordingly prior to study commencement. Interviews were digitally audio-recorded with participants’ permission and were on average 48 minutes in duration (range: 22 minutes—64 minutes). Recordings were transcribed by an experienced external agency and checked for accuracy by the research team.

### Data analysis

Data analysis and management was supported by using NVivo qualitative data analysis software (v11, QSR International Pty Ltd). Data analysis was conducted by interviewers (RAJ and RR) and a female medical student (RP) with experience of psychology research and a background in clinical medicine. Analysis was conducted using a blend of inductive (data-driven) and deductive (theory-driven) approaches as, although the analysis process was guided by the broad research questions, researchers searched for patterns and themes arising directly from the raw data without a-priori expectations or assumptions of the data (DSouza, 2017; Murphy & Dingwall, 2003).

Data analysis was conducted by the lead researcher (RAJ), with two further researchers (RR and RP) coding a subset of transcripts in duplicate (n = 10/20). Duplicate coding helped to maintain reflexivity and reflect on how data was coded, challenge any assumptions made during coding, and identify aspects that may have been missed or overlooked. Researchers remained conscious of their positionality and how this may influence data interpretation. For example, RAJ has lived experience of obesity and mental health diagnoses and has previous experience delivering behavioural weight management interventions. Any inconsistencies between coders were resolved through discussion. The coding framework was developed by RAJ and refined with the support of the wider research team. The wider research team had a background and expertise in psychology, social science, mental health, obesity medicine, and weight management. Researchers involved in data analysis and the wider research team regularly discussed the findings as part of the iterative analysis process.

We used reflexive thematic analysis to identify patterns of meaning across the data by familiarization, coding, theme development, and revision. Reflexive thematic analysis is ideal for analysing data pertinent to individual experiences and/or behaviours, reasons why individuals think or feel or behaviour in such a way, and the factors underpinning and shaping these experiences (Braun & Clarke, 2013, 2019; Virginia Braun & Clarke, 2013). We followed the six phases of analysis described by Braun and Clarke: 1) Familiarization with the data, 2) Coding, 3) Generating initial themes, 4) Developing and reviewing themes, 5) Refining, defining, and naming themes, and 6) Writing up (Braun & Clarke, 2013, 2019, 2021, 2020b; Clarke & Braun, 2017; Virginia Braun & Clarke, 2013). Greater detail of the analytic process can be found in the supplementary material (Supplementary materials two: The analytic process).

Mental health terms, such as anxiety, are both medical terms and part of lay language. We were conscious to use the terms used by participants to describe their experiences, therefore avoiding misidentifying a participant’s mental health. For example, when a participant described symptoms of anxiety (e.g., worry, panic) but did not use the term “anxiety”, we were conscious to not refer to the participant as having or experiencing anxiety. We used medical terms such as anxiety only when a participant had stated this themselves.

### Patient and public involvement (PPI)

Representatives from an established Patient and Public Involvement (PPI) panel were involved in informing trial design and the acceptability of the research questions, interpretation of the results, and dissemination of study findings. The PPI panel included representatives with experience of obesity and weight management. A PPI representative (AV) actively contributed to data analysis and interpretation and critical appraisal of the manuscript, resulting in authorship.

## Results

Four themes were conceptualized from the analysis of the data that address the research questions. Participants are identified with “P” followed by an identification number and their gender and ethnicity (e.g., P1: Female, White).

### Theme 1: Changes in mental health associated with the SWiM-C intervention

#### Mental health improvements associated with the SWiM-C intervention

Participants expressed how being involved with the SWiM-C intervention improved their mental health, for example, by lifting their mood.

“*I felt really happy and positive doing the 12 weeks and I think it sort of … You just sort of … It just put me into a really sort of happy, positive mindset and feel that I was doing something for myself and sort of achieving something for myself”* (P20: Female, Black/African/Caribbean/Black British).

These participants identified that the strategies in the SWiM-C intervention were helpful for their mental health, such as how to manage food cravings, practice willingness, reframe thoughts, and practice breathing techniques.

“*It’s hard to find positive and motivation when you’re in that sort of low mood and feel like everything’s defeating you but when I, you know I sat there sort of ‘wheeer’ and like when I got to the strategies that just, it gave me a little bit of a boost, a little bit of positivity you know, a little bit of light on what negative I was feeling, so yes again it comes down to those strategies I guess*” (P1: Female, White).

Participants characterized the intervention content and coach as compassionate and as providing a level of support that made them feel less alone in the weight management process.

“*It’s like sitting in a room, it felt for me, like sitting in the room with somebody else who was encouraging you to address your weight issues, yeah. Not forcing you, but encouraging you. So it was always like there was someone there*.” (P17: Female, Mixed).

Participants also described how, despite the difficult circumstances of the COVID-19 pandemic, their symptoms of poor mental health reduced since beginning the SWiM-C intervention.

“*My anxiety levels have been sort of up and down, but I would definitely say, since doing the programme [SWiM-C], they’ve definitely been lower and I’m not as anxious*.” (P20: Female, Black/African/Caribbean/Black British).

For participants who experienced weight loss, it was described that this contributed to improved confidence and mood whilst giving them a sense of achievement. In addition, participants directly associated making behavioural changes (such as to their diet and/or exercise) with general improvements in their mental health.

*“I feel good, I feel lighter, my clothing fits better, yeah, I feel more confident in myself, so it’s been a definite improvement*” (P4: Male, White).

“*I can just go and walk the whole circumference of this park, which takes me approximately an hour. So that’s an hour out of my day where I’m breathing fresh air, I see the trees, and I’ve lovely views. And I come home and I feel so much better” … “because I work shifts, I mean, with the time, sometimes I’m away from home for two days, and I miss them. So the first thing that I do when I get home is I do that, just for my own mental health and wellbeing*.” (P19: Female, White).

#### Mental health deterioration associated with the SWiM-C intervention

Participants felt that they were not doing the SWiM-C intervention justice by not doing “enough” or engaging sufficiently (e.g., P5: Female, White), and that they had personal responsibility for lack of results, rather than a limitation with intervention effectiveness. This led to self-blame and negative self-talk.

“*I didn’t seem to be losing weight so then I thought, well I’m not doing what I should be doing, again it’s being down on myself, like I’m not listening enough or I’m not spending enough time … As I say, it’s very much an emotional thing with me so it was kind of stressful in that way*” (P5: Female, White).

Others shared their disappointments in the programme, such as finding the content obvious and “infantilising” (P12: Female, Mixed), resulting in feeling this was another attempt to improve their health and wellbeing that was not going to work.

“*When the obvious is pointed you know, you know you’re really struggling and it gets you down because you can’t get on top of it and control it, then if you read that it just makes you feel like you’re hitting another brick wall, it just makes you feel like you’re not, you know there’s no hope! You just lose heart in it more I think if those things are pushed at you, the things that you know*” (P1: Female, White).

Participants described having to make decisions between competing demands, being the SWiM-C intervention and the other priorities in their lives. They shared that the SWiM-C intervention became “too much” in combination with the other priorities in their lives, and other demands on their “mental energy” (P4: Male, White). Study participants also described the competing demands on their mental energy to lead to deteriorations in their mental health. For example, one participant described increased symptoms of “anxiety” and “stress” (P5: Female, White).

“*If you’re going about your day and you’re busy and you’re trying to sort of focus on losing weight, fine, and you’re incorporating one thing into your life, and it takes mental energy to try and remember one thing, for instance, one little change, to kind of stick to that. And to then every week have to add something on top of that and to think differently and to incorporate something new, it was just too much work. Er … one or two things, you know, where you can get into a routine would be okay, but to try and get everything in was too much*” (P4: Male, White).

### Theme 2: Factors external to the intervention that negatively impacted mental health and intervention engagement

#### Factors external to the intervention that negatively impacted mental health

Participants described experiencing motivational conflicts between SWiM-C and external factors in their lives, as described in Theme 1.2. Participants shared how the external factors in their lives negatively impacted their mental health, including the various ways that the COVID-19 pandemic impacted their lives and resulted in a decline in their mental health. For example, government restrictions (including “stay at home” and shielding orders) resulted in feelings of loneliness. This was exacerbated for those living alone.

“*I live alone as well and I think during, you know, the Covid period, you know, peoples’ mental health goes down because you are alone*” (P17: Female, Mixed)

Other COVID-19 related issues that impacted participants’ mental health included work-related stress (e.g., furloughing, job loss, lack of workplace support, increased workload) and health-related concerns (e.g., worries for self and others risk of contracting COVID-19).

Furthermore, participants expressed feeling “stressed” (e.g., P14: Female, Asian or Asian British) and “depressed” (e.g., P17: Female, Mixed) by the media reports about the pandemic, especially when the media reported on the links between COVID-19 and obesity. In addition, government decisions around restrictions caused stress, frustration, upset, and anger. It appeared that participants’ negative psychological responses to the pandemic and related restrictions commonly stemmed from fear of illness, disagreement with governmental decision making, and feeling that the COVID-19 reporting was “constant” (P17: 61y, Female, Mixed).

“*I felt stress because all the media and kind of reports were saying that if you’re obese then you’re much more likely to have a bad reaction to Covid*” (P14: Female, Asian or Asian British).

“*It’s just so short-sighted for people to just like think it’s okay to get together … it’s just like crazy and he [Boris Johnson, UK Prime Minister] should say nobody can mix. But he won’t … I’m angry. At the moment I’m angry. I get angry really quickly, I’m quite short-tempered which upsets me because I don’t like being like that, but yeah. I do feel like I’m snapping really quickly*” (P3: Female, White).

Additional external factors that negatively impacted participant mental health included feeling unsupported by family members (e.g., disregarding dietary wishes/goals), experiencing health concerns (e.g., arthritis, spinal problems, cancer scares), and experiencing the loss of a family member (e.g., recent loss of their father).

#### Factors external to the intervention that negatively impacted intervention engagement and, consequentially, mental health

Participants who described experiencing motivational conflicts between the SWiM-C intervention and other demands in their lives reported that SWiM-C became the lesser priority for them. These participants described feeling that they should have made more of an effort to engage with the SWiM-C intervention (e.g., by finding more time), but that the intervention “*was an extra thing to try and fit in around everything else*” (P5: Female, White) and did not seem important in comparison with their competing priorities.

“*I was in the midst of all the stress, and I had to make time for it [SWiM-C], but then that is life. In the list of priorities of things, it [SWiM-C] didn’t seem very important at that time*” (P15: Female, Asian or Asian British).

When these participants experienced motivational conflicts and SWiM-C became the lesser priority, they described subsequent reductions in their intervention engagement. The decline in intervention engagement resulted in feelings such as guilt, blame, shame, disappointment, and annoyance at oneself.

“*I think I started okay and then it started building up, the anxiety about it a little bit and because I was so tired, I couldn’t focus enough as well as I wanted to*” (P5: Female, White).

### Theme 3: Use and impact of coping responses

#### Maladaptive coping responses (MCR) and the resulting impact on mental health

Participants described experiencing poor mental health due to a multitude of factors, including those relating to the intervention (see Theme 1: e.g., obvious and infantilising content) and external to the intervention (see Theme 2: e.g., family death, work-related stress). Participants also described experiencing motivational conflicts between the SWiM-C intervention and external factors in their lives, resulting in a decline in their mental health (see Themes 1 and 2).

Participants reported using maladaptive coping responses (MCRs) to manage this deterioration in their mental health. The MCRs described included the use of food to cope, such as comfort eating, binge eating, and eating foods that they perceived to be unhealthy.

“*I think the more stressed I am the worse my diet is. I do tend to binge on sort of sweet things … I do find that when I’m stressed I do binge on sweet things, on chocolate … So, yeah, my mood, or my eating is affected by my mood*” (P4: Male, White).

Conversely, one participant described eating less in response to anxiety or anxiety-like symptoms.

“*I’m not a comfort eater, in fact I stop eating if I’m anxious or frightened or anything like that*” (P18: Female, White).

Participants also described being more sedentary in response to negative emotions (e.g., low mood, stress). For some, this was instead of using a food-based MCR, whilst for others food-based MCRs co-occurred with increased sedentary behaviours and reduced physical activity. Participants described having a lack of “mental energy” (P4: Male, White) to exercise when experiencing negative emotions.

“*I’d be much more inclined to just sit and watch television or something, rather than actually go tuck into food if my mood is down*” (P3: Female, White).

“*I think the more stressed I am the worse my diet is*” … “*that [exercise] can also be affected where, if I’m just not in the head space and if I am stressed, then I don’t have the mental energy to exercise and go to the gym, so that is also affected, yeah*.” (P4: Male, White).

Participants expressed that they used MCRs to compensate for negative emotions, with the aim to improve their immediate mood. However, using MCRs often made them feel worse overall, thus acting like a “vicious circle”. For example, after occasions of using food to cope with negative mood, some participants described being self-critical.

“*If you are in a low mood for quite some time you get out of being good and eating well, and also when you’re not eating well you don’t feel good. So it’s a bit like a knock-on effect. It’s a bit of a vicious circle that if you get into that mood and your eating’s not good, you don’t feel good, you don’t feel, you don’t feel energised, you don’t feel, you look back and you think “well why did I eat that whole Cadbury’s Cream Egg?” or “why did I … ?” and knock yourself down a little bit then. It is a vicious circle*.” (P16: Female, White).

In contrast, one participant did not believe their use of food based MCRs acted like a vicious cycle. They explained that although they snacked more due to work-related stress, they did not eat to compensate for the weight gain due to the stress-snacking.

“*I do tend to snack more … it’s definitely gone up a bit since Covid but as I say that’s just from work stress, but it’s not a feedback spiral, i.e. I’m not snacking more seeing myself gain weight and therefore snack more in order to compensate the stress from that, you know, so it’s linear and not exponential*” (P2: Male, White).

#### Using adaptive, rather than maladaptive, coping responses to manage poor mental health

Participants described that aspects of the SWiM-C intervention supported them to manage the deterioration in their mental health in an adaptive, rather than maladaptive, manner. For example, the intervention strategies and content were reported to be helpful for managing their responses to difficult emotions or moods in a more adaptive manner.

“*It [SWiM-C] certainly, with the mood it does help when you’re, on the days that you’re feeling bleurgh, that you’ve got the strategies to cheer yourself up for want of a better phrase and get yourself to a more positive frame of mind. Whereas in the old days I’d just go and have a six pack of crisps or whatever, it gives me another alternative to say right okay, let’s not do this, let’s look at what else I can do instead, you know” … “it’s more the avoiding the behaviours and how you replace them with other behaviours which are not as destructive to your weight loss regime*” (P7: Male, White).

Participants identified strategies/content that they found helpful to avoid using maladaptive coping strategies. These included content on overcoming obstacles, creating an emotional responses plan of alternative responses, reframing their thoughts, practicing willingness, breathing techniques, and techniques to manage cravings. Content and strategies related to managing cravings, such as urge surfing, were considered particularly useful.

“*You know you feel the urge coming, and think of it as a wave, and actually that, I found that really useful because it reminded me of when I was in labour with my children and thinking that the end would come, although it doesn’t feel like it’s going to now” … “ when I was thinking of the urge of something, you know, it was building up, I was going, ‘here we go, here we go’” … “what I was reading, you know, I was following it, and then I was thinking, ‘right the wave’s going to crash down and disappear now’, and that did help*” (P1: Female, White)

In addition, participants found approaches derived externally to the intervention also helped to respond to difficult thoughts and feelings in a more adaptive manner. These included making use of exercise to improve their mood and pass time whilst experiencing hunger cravings, focusing on little things that brought them joy, and making use of general practitioner (GP)-prescribed sleep medication.

### Theme 4: Preferences for intervention components based on psychological needs, and the desire for intervention personalization

#### Preferences for intervention components based on psychological needs

Participants who described feeling that their weight management problems were emotionally derived and those who experienced fluctuations in their mental health reported finding the psychological content that focused on thoughts and emotions more useful than other content, such as addressing dietary behaviours.

“*It was more the mental side of things. It was more the psychological side of things that was more use to me. So, I know a lot of it dealt with that and the behaviours and the reinforcing and so on and so forth, and the mental work arounds, that bit was helpful*” (P7: Male, White).

Participants specifically noted that SWiM sessions on weight stigma, self-acceptance, and reframing thoughts were most useful, alongside the strategies (e.g., urge surfing) that were included in much of the psychological content. They also expressed a wish for more in-depth psychological content on emotional responses to food and how mood impacts diet, exercise, and sleep.

*“Just the emotional side of it [was most helpful] … yeah I think it could be more in depth on that side of it” … “I think they could do a lot more on that side of it. Just more support on like stress and how it affects your eating, your sleeping, your activeness of doing active things to keep the weight off” … “More support, in a psychologically, sort of why you do it and why, more than the basics. And ways of sort of helping you emotionally cope better with food.” (P11: Female, White)*

Similarly, participants who believed that their weight management problems were emotionally derived described wanting more regular contact with the SWiM coach. Participants described wanting different things from this contact, including counselling-like support, a general check-in, or a safety/welfare check. They felt that additional contact with the coach would help them by having personal human contact, an opportunity to ask nuanced questions, and support to stay engaged during emotionally difficult times.

“*You can tell people what to eat but you, we know, it’s like telling an alcoholic isn’t it, don’t drink, but, and I think it’s more … we know what we should be eating but there’s a problem, emotional around food. So I think there needs to be more counselling, psychological to help and support for the individual who’s struggling*” (P11: Female, White)

“*To be honest I know it would be more work, but I would even perhaps throw extra calls in, so rather than just the one call maybe two or three spread throughout the course. Because then if I had hit a buffer for example, because of my dad dying I hadn’t done it for a couple of weeks, I would have been able to be picked-up a little bit quicker than I was, and maybe alternative arrangements made at that point*” (P7: Male, White)

Participants who described not experiencing mood fluctuations or poor mental health noted that they did not find the psychological content useful as it was not relevant or applicable to their needs. These participants saw little benefit from the content on stress, self-acceptance, emotional eating, weight stigma, and depression.

“*I was more interested in the steps and strategies than the emotional wellbeing sides of it because I don’t really think … I think I’m more half-full than half-empty as a general thing and I don’t think, I know some people struggled through this last year, I don’t think I’m one of those. So I don’t think low mood is a particular feature for me, or anxiety or anything like that*” (P8: Female, White)

#### The desire for intervention personalization, and awareness of the limitations and risks

Participants felt that the intervention was too restrictive and that it “penalised” (P16: Female, White) them by having to complete a session before unlocking the next, meaning the participant was unable to skip sessions they were not interested in. As a result, participants expressed an interest in the intervention being tailored to their individual psychological needs, with a potential needs-assessment call or questionnaire at the beginning of the intervention to determine the type of session content, and the frequency and regularity of coach contact, that they may require. This could allow participants to select more or less psychological content dependent on their perceived individual psychological needs.

“*It was asking all sorts of things, “Are you stressed with this, are you stressed with that? Do you have family stresses?” I don’t. I mean, you know, I was sort of going through it, I mean, it was just all, all no, no, no, no, no for me. I mean, I understand that everyone’s different. Erm … it’s, I suppose at the start you’ve got to just assess what sort of type of course they’ve got to be on*” (P6: Male, White)

Participants suggested that intervention personalization could be achieved by ordering the sessions in different ways, adding or removing particular sessions, and changing the amount of SWiM coach contact.

“*I think it’s quite helpful for people to think about where they are, and why they are there at the beginning maybe … Because I think then what you could do is you could direct people, I mean but you could say, you know, you might help, find it helpful to do things in this order perhaps*” (P18: Female, White)

Participants were aware of the potential limitations and risks of intervention personalization, recognizing that an intervention cannot suit everyone’s needs and that there will always be intervention component(s) that are less relevant to some. Participants were aware that intervention personalization depended on resource limitations, such as SWiM coach capacity, and recognized that it may be challenging to personalize an intervention based on psychological needs, as these can change regularly. Finally, participants were also aware that intervention personalization risked a participant choosing to not complete a session due to failing to recognize the relevance of this content for them.

“*I mean obviously you don’t want it to be creating the opportunity to put somebody to be in denial and skip something which really is for them*” (P18: Female, White)

## Discussion

The purpose of this qualitative study was to broadly explore participants’ mental health experiences during the SWiM-C intervention, with the aim of understanding how SWiM-C and other weight management interventions could be adapted to better support mental health. We found that participants were exposed to a range of experiences related to (see Theme 1) and external to (see Theme 2) the SWiM-C intervention that impacted their mental health. Many participants shared that they experienced improvements in their mental health during the intervention. However, some felt that the intervention was mentally draining and increased their stress. A recent systematic review by the study authors found that behavioural weight management interventions, on average, result in small benefits for mental health (Jones et al., 2021). It is notable that the current study identifies varied experiences of the impacts on mental health. This highlights that, although the average effect may be positive, a proportion of participants do experience a decline in their mental health that they attribute at least in part to participation in the intervention.

Previous research has reported that weight management efforts require a vast amount of mental and emotional investment (Kirk et al., 2014); the authors did not define “investment”. We infer that this suggests weight management efforts can be draining, aligning with the findings of the current study. Participants perceived to make trade-offs between the SWiM-C intervention and the external factors in their lives, including work-related stressors, negative impacts of the pandemic, and life crises (e.g., grief). Study findings show that participants perceive trade-offs between the competing demands as feeling “too much” and taking “mental energy” (e.g., P4). This may link to the psychological theory of motivational conflicts and ego depletion. This literature describes competing demands on mental energy as “motivational conflicts”, and the resultant draining of mental energy and feelings of overwhelm as “ego depletion” (Baumeister & Vohs, 2007; Hayward et al., 2018; Tice et al., 2007). When in a state of ego depletion, it may be harder to make choices in line with our goals and values (Baumeister & Vohs, 2007; Hayward et al., 2018; Tice et al., 2007). The participants of this study described that they often experienced the SWiM-C intervention as of lesser importance when faced with competing priorities, thus making a choice that was not in line with their weight management goals and values (see Theme 2). Participants described the resultant decline in intervention engagement to lead to feeling self-blame, shame, guilt, and disappointment in themselves (see Theme 2), which might further deplete mental energy.

The findings of this study have suggested that participants may experience motivational conflicts between the SWiM-C intervention and the external demands in their lives, leading to diminished mental energy and a state of ego depletion. Research shows that ego depletion is associated with a decline in mental wellbeing and an increased likelihood of using maladaptive coping responses (MCRs), with the use of MCRs further worsening mental health (Baumeister & Vohs, 2007; Hayward et al., 2018; Tice et al., 2007). Our study findings align with this literature; we found that participants described using maladaptive coping responses to manage the negative consequences from balancing the competing demands on their mental energy. Our findings highlighted that both managing the competing demands and the demands themselves negatively impacted their mental health. Participants described how they hoped these coping responses would improve their mood, but that often their mental health was further damaged via feelings of regret, self-blame, and shame (see Theme 3). This is consistent with literature reporting that use of MCR in adults with overweight or obesity was associated with poorer psychological wellbeing (Hayward et al., 2018). This further supports the study findings that use of MCR exacerbates the decline in participants’ mental health, rather than mitigating the existing decline.

Reducing the likelihood that participants manage worsening mental health with MCRs is important to minimize any further psychological harm. Previous research has suggested that positive affect and mental rest can counteract the effects of “ego depletion” (i.e., worsening in mental wellbeing and use of MCRs) (Hayward et al., 2018; Tice et al., 2007). In this study, we found that aspects of the SWiM-C intervention appeared to help participants to respond adaptively, rather than maladaptively (see Theme 3). For example, participants felt the compassion of their coach and the supportiveness of the intervention content improved their mental health, whilst intervention strategies, such as reframing thoughts and breathing techniques, helped to avoid using MCRs. Similarly, Frayn et al. found that participants endorsed the use of alternative stress reduction and coping strategies (e.g., targeting emotional regulation) to mitigate the effects of using food-based MCRs, such as emotional eating, aligning with the findings of this study (Frayn et al., 2018). It is possible that the SWiM-C intervention counteracted the effects of ego depletion via improvements in positive affect and mental rest, however further research is required to explore this hypothesis. Future trials of weight management interventions may consider including more strategies that target emotional regulation to investigate whether a potential reduction in use of MCRs occurs.

In this study, participants also shared how they believed the current intervention could be adapted to better attend to their psychological needs (see Theme 4). Those who experienced poor mental health expressed a preference for more in-depth psychological content (e.g., content on emotional eating or stress management) and a greater amount of coach contact. Conversely, those who felt they did not experience fluctuations in mood were happy with the frequency of coach contact and expressed a preference to opt out of psychological content. Participants suggested this could be achieved through intervention personalization, however they recognized that this may risk participants opting out of a session that could benefit them. A recent systematic review synthesized evidence on tailored eHealth interventions for weight loss and found only six digital weight loss interventions to be personalized according to participant characteristics (Ryan et al., 2019). Our study findings, alongside this lack of evidence, suggests that researchers may consider incorporating personalization into future trials of weight management interventions. Researchers may achieve this by making all intervention content available at baseline, rather than participants having to complete a session before “unlocking” the next. This would allow participants the flexibility to navigate through the content in an order and pace appealing to their individual wants and needs.

A recent systematic review found behavioural weight management interventions based on acceptance and commitment therapy (ACT) to be effective for weight loss, with evidence to suggest that these interventions may be more supportive of mental health than standard behavioural treatment (Lawlor, Islam et al., 2020). The findings of this qualitative study align with those of the systematic review as we highlight the potential of ACT-based interventions to psychologically support adults with obesity during weight management. Specifically, we identified that intervention strategies with an ACT focus (e.g., reframing of thoughts, cognitive defusion, and urge surfing) were beneficial for mental health in some participants. In addition, these ACT-based strategies supported many participants to respond to declining mental health in an adaptive manner (e.g., visually the urge, using an emotional response plan) when they previously responded maladaptively (e.g., binge or comfort eating). The findings of this study add to the evidence base and strengthen our understanding of how ACT-based weight management interventions can support mental health.

The findings of this qualitative study represent an important contribution to understanding the role of mental health in behavioural weight management interventions for adults with overweight or obesity. This is of particular value due to the limited number of studies focusing on participants’ mental health experiences during weight management interventions. There are several implications to be considered. Firstly, we suggest that intervention developers should consult with patient representatives to minimize participant burden, especially as our findings suggest that participants manage many competing demands that are experienced as draining their mental energy, making them feel overwhelmed, and may thereby negatively impact mental health. Intervention developers may wish to consider including strategies/content on emotional regulation to support participants to respond adaptively, rather than maladaptively, when experiencing poor mental health during weight management interventions. Further, intervention developers may wish to consider integrating personalization within scalable behavioural interventions to support participants to tailor the content according to their individual psychological needs. Finally, healthcare practitioners providing services may consider talking with a participant about the concurrent demands in their life that may impact their mental energy and mental health. By doing so, the participant and practitioner may jointly reflect on whether this is an appropriate time to begin the intervention as it will result in further demands in their lives. The recently published guidance for healthcare practitioners on conversations about obesity may support these interactions to be non-stigmatizing (Crotty & European Coalition for People Living with Obesity (EPCO), 2021; McGowan, 2016).

### Limitations of the study

It is possible that study participants were subjected to more demands that required mental energy and impacted their mental health more than usual due to the circumstances of the pandemic. As a consequence, the intervention may have been a lesser priority during the study period than it may have been outside of the circumstances of the pandemic. Although the pandemic represents a unique set of circumstances, findings may be transferable to other contexts of heightened stress and distress. Future research may wish to assess whether the intervention is differentially prioritized during times of increased and decreased stress and distress. This understanding may inform the design of intervention recruitment and engagement strategies in the future.

A concern in qualitative research, particularly when addressing potentially sensitive topics such as mental health, is participants feeling vulnerable sharing their experiences. We made conscious efforts to support participants to feel safe to share their experiences, including use of a compassionately grounded interview style (e.g., offering reassurance when appropriate), contacting the participant prior to the interview to increase familiarity, and clearly communicating their rights to withdraw as well as the terms of confidentiality (Elmir et al., 2011).

We also recognize that assigning data to themes requires assessment of interpretation and meaning and, as with all qualitative research, researcher positionalities can influence assessment of data and the development of study findings. The potential bias due to the subjective nature of qualitative research was minimized by regular discussion between authors (including a patient representative who participated in SWiM-C intervention), second coding of interview transcripts, and iterative development of the framework.

Furthermore, we acknowledge that interpretation of language can differ greatly, particularly as mental health terms exist in both medical practice and lay language. For example, the term “mental health” can be defined and interpreted in multiple ways. We were conscious to appropriately represent participants’ mental health by using their language, therefore minimizing the risk of misdiagnosing or misrepresenting a participant’s mental health.

In this study, we recruited participants for the maximum variation in demographic characteristics, strengthening the transferability of findings. However, we appreciate that the findings may not be applicable to all adults with overweight or obesity engaged with an online behavioural weight management intervention. Specifically, our sample reflects the population of the parent trial, which was largely made up of White, highly educated, females. Future research should consider exploring the mental health experiences of more specific participant groups (e.g., by age, gender, presence of mental health/disordered eating diagnosis) to compare findings.

## Conclusion

In this study, we provide an in-depth examination of participants’ mental health experiences during a web-based, guided self-help, ACT-based intervention during the COVID-19 pandemic. We found that many SWiM-C participants were exposed to a range of experiences, both related to and external to the intervention, that negatively impacted their mental health. We found participants used MCRs with the aim to manage these challenges and improve their mood, however the MCRs more often resulted in further decline in psychological wellbeing. Notably, aspects of the SWiM-C intervention (e.g., intervention strategies, such as reframing thoughts and breathing techniques) appeared to support some participants to manage their mental health more adaptively. Intervention personalization based on psychological needs was suggested to better support participant mental health. The findings can be used to inform the development of future weight management interventions, as well as informing how SWiM-C could be adapted and optimized for other contexts. Future trials may wish to explore the influence of intervention personalization and the inclusion of more strategies that target emotional regulation.

## Supplementary Material

Supplemental Material

## Data Availability

The dataset analysed during the current study is not publicly available. Participant consent allows for data to be shared in future analyses with appropriate ethical approval, and the host institution have an access policy (https://www.mrc-epid.cam.ac.uk/wp-content/uploads/2019/02/Data-Access-Sharing-Policy-v1-0_FINAL.pdf) so that interested parties can obtain the data for replication or other research purposes that are ethically approved. Data access is available from the senior author, who is also the principal investigator of the SWiM-C trial, upon reasonable request (ala34@cam.ac.uk).
