# Supplementary material for: Participants' experiences of mental health during a COVID-19 tailored ACT-based behavioural weight management intervention: a qualitative study
Source: Int J Qual Stud Health Well-being. 2022 Sep 13;17(1):2123093. doi: 10.1080/17482631.2022.2123093 (PMC9481081; doi:10.1080/17482631.2022.2123093)
Supplement: Supplemental Material [file ZQHW_A_2123093_SM4837.docx]

**Supplementary material one:** **Interview schedule for intervention participants**

| **Weight loss journey**   1. Before we talk about the [Trial name redacted for blinding] programme, can you briefly describe your weight management journey before beginning the programme? 2. People join these programmes for many different reasons.   Can you tell me what were your main motivations for joining the [Trial name redacted for blinding] programme?   1. What were your hopes or expectations for the programme?    1. To what extent do you feel your hopes/expectations were met?   **Programme acceptability**   1. How did you find using [Trial name redacted for blinding] website?    1. Prompt: How easy or difficult and why, any technical issues, finding the time/privacy 2. Can you tell me about the information delivered in the [Trial name redacted for blinding] sessions?    1. What did you find most useful?    2. What did you find least useful?    3. Is there anything you would have liked to have more support with?    4. Is there anything you feel is missing from the programme?    5. Is there anything you feel shouldn’t be included in the programme? 3. The programme provided information on skills and strategies for weight management and emotional well-being.    1. How easy or difficult did you find it to understand the strategies or exercises? Did anything help or hinder your understanding?    2. Which strategies, exercises or [Trial name redacted for blinding] Aids did you find most useful?    3. Which strategies, exercises or [Trial name redacted for blinding] Aids did you find least useful? 4. How useful did you find your conversations with the coach?    1. What did you find most useful?    2. What did you find least useful?    3. Is there anything you would have liked to have more support with?   **Benefits and disadvantages**   1. Can you tell me about any positive experiences, or benefits, you have experienced as a result of the [Trial name redacted for blinding] programme? 2. Can you tell me about any negative experiences, or difficulties, you have experienced as a result of the [Trial name redacted for blinding] programme?   **Mental health/emotional well-being**   1. I’d be interested to know more about what your mood was like during the programme? 2. For example, to what extent have you experienced…    - 1. feelings of stress?      2. conflict at home/at work?      3. financial concerns?      4. loneliness?      5. feeling not sufficiently supported?      6. not feeling fully satisfied with your life? 3. In what ways do you feel that your mood affects your health behaviours, such as your diet, movement, or sleep? For example, if you are feeling low, stressed, lonely, or having a bad day? 4. How is your diet affected? How is your physical activity affected? How is your sleep affected? 5. To what extent did participating in the [Trial name redacted for blinding] programme affect your mood and emotions? How? Why?    1. Did the programme provide sufficient support?    2. What additional support do you think may be helpful? Anything else? 6. Before I ask my final question, I’m going to ask you to take a moment to reflect on your time taking part in the [Trial name redacted for blinding] programme. When you reflect back on your time as a [Trial name redacted for blinding] participant, can you describe kind of feelings emerge for you? Anything else? |
| --- |

**Supplementary material two: The analytic process.**

| **Phase** | **What this looked like** |
| --- | --- |
| **Phase one: Familiarisation with the data** | We achieved deep familiarity with the content of the data by immersing ourselves in the dataset. This involved listening to each of the transcripts from start to finish and, separately, reading all transcripts from start to finish. Whilst listening/reading, we made hand-written doodles and scribbles relating to things that stood out.  During and after listening/reading each transcript, we practiced reflexivity by reflecting and asking ourselves deeper questions about the data. Examples of questions were: Would I feel similar to the participant if experiencing similar things (and why might this be similar/different)? What am I feeling, and why might I be reacting in this way? Why might I be interpreting the data in this way, and what other ways could this be interpreted?  During this phase, we noted particularly interesting or intriguing elements that stood out and identified multiple points of potential analytic interest (e.g., the impact of SWiM-C on participants mood). Although the process of familiarisation was benefited from a rich understanding of the topic, critical self-inquiry (i.e., practicing reflexivity) aided inspection of positionality influences on interpretations. |
| **Phase two: Coding** | Each coder reviewed each interview transcript and applied code labels to segments of data related to mental health. All data related to mental health were coded as the research question was broad and exploratory. Some data were not coded as they were not related to the broad research interest, being mental health. A few hundred codes were initially produced. On the spectrum of sematic (i.e., explicit and surface meanings) to latent (i.e., interpretative), the codes applied were of more latent orientation as we sought to interpret, rather than simply describe, the data. Sematic elements were maintained when participants used mental health language (e.g., anxiety) to avoid misdiagnosing or misrepresenting the participants’ experience. All data coders made an active effort to resist thinking about themes during this stage as this can result in cherry-picking and foreclosing analysis (i.e., ceasing analysis after only superficial engagement with the data).  The leader data coder coded four transcripts before meeting with second coders to compare and discuss code labels. We had in-depth discussions about how we interpreted the data, why we had made particular decisions, and how our positionality may have influenced our interpretations. We did not aim for consensus, rather used this process with the aim of “*developing richer and more complex insights*” (Braun and Clarke, 2021: Page 55 – Thematic Analysis: A Practical Guide). |
| **Phase three: Generating initial themes** | Each coder engaged with the data codes to explore clusters and patterns of meaning across the dataset. Each coder independently developed an early thematic map that represented proposed candidate themes (i.e., potential broad patterns of meaning). We met to compare early thematic maps - we critically discussed our maps and questioned each other on why we had made particular decisions, how we had interpreted the data, and what may have influenced our interpretations. This process of challenging one another’s assumptions and interpretations supported the practice of reflexivity. The thematic map was revised based on these conversations. |
| **Phase four: Developing and reviewing themes** | This phase aimed to review the viability of the themes and refine them accordingly. We reengaged with the coded data extracts related to each theme and asked questions about the codes, coded data, and themes. For example:   - Does the theme have a central concept and include multiple dimensions of that idea? - Does the theme have clear boundaries (what should and should not be included)? - Is there enough supporting data and is it meaningful enough to support the theme? - Is the theme coherent, or is the data too diverse and wide-ranging? - Does the theme communicate something important, meaningful, and relevant to the overall question?   The lead data coder also reengaged with the entire dataset by reviewing each interview transcript again from start to finish to further refine the code labels and apply new code labels to any data that was overlooked/missed during phase two (coding). It was important to return to the entire dataset at this point in the analysis as it is possible to become distant and a few steps removed from the data during theme development, and reengagement with the data reduces the likelihood of misrepresenting the dataset. We remained flexible with the thematic map during this stage, allowing for further refinement of themes and code labels.  Reengagement with the coded extracts and entire dataset allowed us to develop, review, and refine the themes. The refined themes (and associated code labels) were circulated to the authorship team to discuss their relevancy to the broad question, legitimacy and meaningfulness as themes, and the overall flow/narrative. |
| **Phase five: Refining, defining, and naming themes** | We explored the dataset for contradictions within transcripts and across the dataset. We defined and named the themes with a short phrase that captured the central concept. We circulated the refined thematic map (and associated code labels) to the authorship team for feedback and discussion. We described the thematic map (themes and overall narrative) to multiple people outside of the authorship team to test whether the scope, boundaries, and core concepts of the themes were sufficiently developed, and whether the overall narrative had a logical and meaningful flow.  There was back and forth movement between phases three, four, and five until there was a final set of themes and related coded extracts. |
| **Phase six: Writing up** | The write-up provided a final opportunity for refinement of the themes. We selected data extracts from across the dataset that provided vivid, clear, concise examples that supported the analytic claims. After writing up the analysis, we again reflected on the logical flow of the narrative and the ordering of the themes. We took a short break from the analysis and write-up to ‘step away’ from the themes, allowing us to return to the results with a ‘fresh mind’ to assess the story across the themes. We drew initial conclusions about the data and analysis, existing knowledge in the field, and the wider context.  This phase involved many rounds of edits, with feedback and suggestions from the authorship team. The aim was not to achieve consensus, instead the aim instead was to achieve rich, meaningful, and impactful conclusions that were well supported by the themes (and associated coded extracts). The overall aim of writing-up was to present a critical exploration of the meanings related to participant experiences of mental health during the intervention, whilst respecting and appropriately representing the participant voice. |
